# Congenital anomalies prevalent in rural population of Dera Ismail Khan, Pakistan: Ethnic inequalities and biodemographic attributes

**DOI:** 10.12669/pjms.42.2.12330

**Published:** 2026-02

**Authors:** Muhammad Asghar Khan, Qumar Zaman, Saima Naz, Azmatullah, Zahida Parveen, Sajid Malik

**Affiliations:** 1 Muhammad Asghar Khan Human Genetics Program, Department of Zoology, Quaid-i-Azam University, Islamabad, Pakistan; 2 Qumar Zaman Human Genetics Program, Department of Zoology, Quaid-i-Azam University, Islamabad, Pakistan; 3 Saima Naz Department of Anatomy, Rawalpindi Medical University, Rawalpindi, Pakistan; 4 Azmatullah Human Genetics Program, Department of Zoology, Quaid-i-Azam University, Islamabad, Pakistan; 5 Zahida Parveen Human Genetics Program, Department of Zoology, Quaid-i-Azam University, Islamabad, Pakistan; 6 Sajid Malik Human Genetics Program, Department of Zoology, Quaid-i-Azam University, Islamabad, Pakistan

**Keywords:** Birth defects, Consanguinity, Descriptive epidemiology, Hereditary anomalies, Neuromuscular defects, Neurological disorders

## Abstract

**Objectives:**

Congenital anomalies (CA) have a high prevalence in Pakistan, but regional and ethnic differences remain less appreciated. This study was aimed to observe the pattern and ethno-demographic distribution of CA in the extended Dera Ismail Khan region of Pakistan.

**Methodology:**

In a descriptive clinico-epidemiological study, families/subjects with CA were recruited from a variety of sources, including district headquarters hospitals, community centers, rural organizations, and door-to-door surveys. Phenotypic and biodemographic data were recorded. Descriptive statistics was applied. This study was conducted from June 2022 to May 2024.

**Results:**

This study included 637 independent individuals (73% males) with certain types of CA. Pediatric patients were 70% of the sample; the majority originated from rural areas (71%) and Saraiki background (88%) and belonged to Jaat and Pathan ethnicities (40%). CA were classified into nine major and 90 minor entities. Among major categories, neuromuscular disorders were most frequent (32%), followed by neurological disorders (24%), sensorineural/ear defects (15%), limb defects (9%), eye/visual impairments (6%) musculoskeletal defects (4%), blood disorders (4%), ectodermal anomalies (3%), and others (4%). Sporadic occurrence was witnessed in 51% of the cases and parental consanguinity in 71%. There were statistically significant differences in the distribution of major categories of CA with respect to rural/urban origin, mother tongue, and ethnicities.

**Conclusion:**

Neuromuscular and neurological disorders, and sensorineural/ear defects had the highest burden in the study population and also showed remarkable disparities across various ethnic groups. The impact of such defects can be reduced through improved health, education, antenatal and prenatal care, pre-marital counseling, and molecular diagnosis of CA.

## INTRODUCTION

Congenital anomalies (CA) or birth defects are abnormalities detected at birth, including functional, structural, metabolic, and behavioral defects.^1^,^2^ CA are typically categorized as minor and major anomalies. A minor anomaly has a minimal clinical impact at birth, whereas a major CA is a severe condition that reduces life expectancy or compromises normal function, potentially leading to stillbirth or infant death.^1^,^2^

Globally, approximately three million infants with major CA are born annually.^1^,^3^ Additionally, an estimated 303,000 neonates die every year within the first month of life worldwide. CA have a severe impact in low and middle income countries, where 95% of affected children die from these anomalies.^2^ The most prevalent categories of CA worldwide include the central nervous system (CNS), cardiovascular system, multiple system anomalies, renal defects, facial abnormalities, and other anomalies, including gastrointestinal, respiratory, urogenital, and skeletal defects.^1^-^3^

The prevalence of CA in Pakistan is very high, although it varies greatly across regions.^4^,^5^ A study conducted in Okara, Pakistan, reported that the high prevalence of CA, with limb defects being the most common, followed by neurological disorders.^5^ Another study conducted in the population of Hazara, Pakistan, reported a high occurrence of neurological disorders and limb defects, followed by musculoskeletal, sensorineural, and blood disorders.^6^ The increased prevalence of CA in Pakistan is attributed to several risk factors, including consanguinity, maternal age, rural origin, low socioeconomic factors, maternal infections, and chemical exposure.^7^,^8^ This study was conducted to address the lack of epidemiological data on CA in the underserved Dear Ismail Khan (DIK) region of Khyber Pakhtunkhwa, where high consanguinity, limited healthcare access, and socio-economic disadvantage likely shape disease patterns but remain poorly documented.

## METHODOLOGY

In this descriptive cross-sectional study, mixed sampling methods were utilized for the recruitment of subjects/families with CA, including data collection from district headquarters hospitals, basic health units, rural support organizations, special education centers, rehabilitation and community centers. Door-to-door surveys were also conducted in remote and rural areas. This approach ensured a more diverse and representative sample from both urban and rural suburbs. Participants were included regardless of sex, ethnicity, or type of anomaly. Only individuals with congenital and/or hereditary malformations were included, whereas cases with accidental or likely infectious origins were excluded. This study was carried out from June 2022 to May 2024.

### Ethical Approval:

The study was approved by the Ethical Review Committee of Quaid-i-Azam University (DAS-19-, July 3, 2019). All information was collected and recorded in the presence of a guardian or family head after participants provided written or verbal consent if they were unable to read. Written consent was obtained from parents, guardians, or literate elders for individuals who were underage or unable to provide consent due to a disability.

### Classification of anomalies and statistical analysis:

All index cases (defined as the first identified affected individual from each family/pedigree) were physically examined and diagnosed by specialized doctors. Individuals from rural areas were taken to the nearest medical center or district hospital for clinical examinations. Pre-diagnosed cases were also included from disability and rehabilitation centers. For statistical analyses, only one index case per family was included to maintain independence. CA were categorized based on the involvement of major organ systems, with definitions adopted from the OMIM (www.omim.org/) databases and ICD-10 (https://icd.who.int/browse10/2019/en#/V). A detailed pedigree extending to three generations was drawn for each patient, and from each family, only the index patient was included in the statistical analysis.

### Statistical analysis:

Statistical analysis was performed to summarize categorical variables. Chi-square and Fisher’s exact tests were used to assess the significance of the distributions, with the significance level set at *P*<0.05. The proportions and 95% confidence intervals (95% CI) were calculated based on the total number of anomalies.

## RESULTS

### Sample characteristics:

In the current study, 637 index cases (468 males and 169 females) with CA from independent families were recruited. Patients aged up to 19 years comprised 70% of the sample; 71% originated from rural areas, 88% belonged to Saraiki-speaking families, 59% had extended families, and 39% were from poor/low socio-economic quartiles.

### Classification of congenital anomalies:

The CA were classified into nine major and 90 minor categories (Tables-I and II). The major categories included neuromuscular disorders (32%), followed by neurological disorders (24%), sensorineural/ear defects (15%), limb defects (9%), eye/visual impairments (6%), musculoskeletal and blood disorders (4% each), ectodermal anomalies (3%), and others (4%; included rare anomalies) (Table-I).

**Table-I:** Major categories of congenital anomalies with respect to gender of index cases.

Major Division#	Male	Female	Total	Proportion	95%CI
Neuromuscular disorders	148 (72)	58 (28)	206 (32)	0.323	0.287-0.360
Neurological disorders	116 (77)	35 (23)	151 (24)	0.237	0.204-0.270
Sensorineural/ear defects	71 (76)	22 (24)	93 (15)	0.146	0.119-0.173
Limb defects	36 (63)	21 (37)	57 (9)	0.089	0.067-0.112
Eye/visual impairments	26 (68)	12 (32)	38 (6)	0.060	0.041-0.078
Musculoskeletal defects	23 (85)	4 (15)	27 (4)	0.042	0.027-0.058
Blood disorders	14 (61)	9 (39)	23 (4)	0.036	0.022-0.051
Ectodermal anomalies	15 (79)	4 (21)	19 (3)	0.030	0.017-0.043
Others	19 (83)	4 (17)	23 (4)	0.036	0.022-0.051
Total	468 (73)	169 (27)	637 (100)	1.000	0.287-0.360
	c^2^=10.20, P=0.25				

#, values presented in numbers and (%).

**Table-II:** Major and minor categories of congenital anomalies.

Major/ Minor Division	Freq. n	Proportion	95% CI	ICD-10	OMIM
***Neuromuscular disorders***	206	0.323	0.287-0.360		
**Cerebral palsy types**	151	0.237	0.204-0.270		
**Spastic types**	60	0.094	0.072-0.117		
Quadriplegia	23	0.036	0.022-0.051	G80.0	
Hemiplegia(left)	12	0.019	0.008-0.029		
Diplegia	9	0.014	0.005-0.023	G80.1	270600
Hemiplegia(right)	7	0.0011	0.003-0.019		
Paraplegia	6	0.009	0.002-0.017		
Triplegia	2	0.003	-0.001-0.007		
Monoplegia	1	0.002	-0.002-0.005		
**Athetoid types**	58	0.091	0.069-0.113		
Athetosis	37	0.058	0.040-0.076		
Dystonia	21	0.033	0.019-0.047	G80.3	
**Ataxic types**	19	0.030	0.017-0.043	G80.4	605388
**CP (unspecified)**	14	0.022	0.011-0.033	G80.9	605388
Lower limb hypotonia	20	0.031	0.018-0.045	P94.2	300868
Muscular dystrophy	18	0.028	0.015-0.041	G71.0	310200
Muscle hypotonia	6	0.009	0.002-0.017	P94.2	
Primary dystonia	5	0.008	0.001-0.015		
Ataxia (unspecified)	3	0.005	-0.001-0.010	R27.0	160120
Duchenne muscular dystrophy	3	0.005	-0.001-0.010	G71.01	310200
***Neurological disorders***	151	0.237	0.204-0.270		
Intellectual disability, ID types	108	0.170	0.140-0.199	F70-F79	300243
ID, mild	20	0.031	0.018-0.045	F70	249500
ID, moderate	25	0.039	0.024-0.054	F71	
ID, severe	19	0.030	0.017-0.043	F72	251200
ID, unspecified	44	0.069	0.049-0.089	F79	
Microcephaly	14	0.022	0.011-0.033	Q02	251200
Down syndrome	7	0.011	0.003-0.019	Q90	190685
Epilepsy	5	0.008	0.001-0.015	G40	117100
Global developmental delay	4	0.006	0.000-0.012	Z13.42	618330
Spina bifida	3	0.005	-0.001-0.010	Q05	182940
Huntington chorea	2	0.003	-0.001-0.007	G10	143100
Hydrocephalous	2	0.003	-0.001-0.007	G91.9	236600
Myelomeningocele	2	0.003	-0.001-0.007	Q05.9	182940
Migraine	2	0.003	-0.001-0.007	G43	
Cerebellar atrophy	1	0.002			618501
Edwards Syndrome	1	0.002	-0.002-0.005	Q91.3	601161
***Sensorineural/ear defects***	93	0.146	0.119-0.173		
Deaf and mute	82	0.129	0.103-0.155	H91.3	304500
Mute only	4	0.006	0.000-0.012	F94.0	
Deaf only	3	0.005	-0.001-0.010	H91.3	
Microtia	3	0.005	-0.001-0.010	Q17.2	600674
Stuttering	1	0.002	-0.002-0.005	F98. 5	184450
***Limb defects***	57	0.089	0.067-0.112		
Talipes types	20	0.031	0.018-0.045	Q66.0	119800
Polydactyly types	14	0.022	0.011-0.033	Q69. 9	
Syndactyly types	4	0.006	0.000-0.012	Q70	609815
Brachydactyly	3	0.005	-0.001-0.010	Q68.81	113000
Bifid thumb	2	0.003	-0.001-0.007		
Cenani-Lenz syndactyly	2	0.003	-0.001-0.007	Q78.4	212780
Radial hemimelia	2	0.003	-0.001-0.007	Q73.8	275220
Split-hand-foot-anomaly	2	0.003	-0.001-0.007	Q72.7	183600
Thumb aplasia	2	0.003	-0.001-0.007		
Amputation	1	0.002	-0.002-0.005	Q73.0	217100
Camptodactyly	1	0.002	-0.002-0.005	Q74.0	114200
Giant feet	1	0.002	-0.002-0.005		
Symbrachydactyly	1	0.002	-0.002-0.005		610713
Thumb hypoplasia	1	0.002	-0.002-0.005	Q06. 1	
Triphalangeal thumb	1	0.002	-0.002-0.005	Q69.1	174500
***Eye/visual impairments***	38	0.060	0.041-0.078		
Blindness	15	0.024	0.012-0.035	H54	216900
Myopia	5	0.008	0.001-0.015	H52.1	311000
Retinitis pigmentosa	5	0.008	0.001-0.015	H35.52	613731
Anophthalmia	3	0.005	-0.001-0.010	Q11.2	251600
Glaucoma	3	0.005	-0.001-0.010	Q15.0	231300
Squint eyes	2	0.003	-0.001-0.007	H50.9	185100
Anisocoria	1	0.002	-0.002-0.005	H57.02	106240
Cataract	1	0.002	-0.002-0.005	H26. 9	
Day blindness	1	0.002	-0.002-0.005	H53.11	
Night blindness	1	0.002	-0.002-0.005	H53.60	310500
Ptosis	1	0.002	-0.002-0.005	H02. 4	178300
***Musculoskeletal defects***	27	0.042	0.027-0.058		
Achondroplasia	5	0.008	0.001-0.015	Q77.4	100800
Skeletal dysplasia	5	0.008	0.001-0.015	Q79. 9	618870
Kyphoscoliosis	3	0.005	-0.001-0.010	M40	610170
Mucopolysaccharidosis	3	0.005	-0.001-0.010	E76.3	252800
Arthrogryposis	2	0.003	-0.001-0.007	Q74.3	108120
Dwarfism	1	0.002	-0.002-0.005	E34.3	100800
Joint hypermobility	1	0.002	-0.002-0.005	M35.7	
Laron syndrome	1	0.002	-0.002-0.005	E34.3	262500
Madlung deformity	1	0.002	-0.002-0.005	LB90. 4	127300
Nail patella	1	0.002	-0.002-0.005	Q87.2	161200
Proportionate dwarfism	1	0.002	-0.002-0.005	Q87.1	223550
Pseudo achondroplasia	1	0.002	-0.002-0.005	Q77.8	177170
Recurrent dislocation of foot	1	0.002	-0.002-0.005	M24.47	
Vit-D resistant rickets	1	0.002	-0.002-0.005	E83.3	277440
***Blood disorders***	23	0.036	0.022-0.051		
Thalassemia major	21	0.033	0.019-0.047	D56	613985
Hemophilia	1	0.002	-0.002-0.005	D66	306700
Myelodysplastic syndromes	1	0.002	-0.002-0.005	D46.9	614286
***Ectodermal anomalies***	19	0.030	0.017-0.043		
Albinism	3	0.005	-0.001-0.010	E70.3	203100
Ichthyosis	3	0.005	-0.001-0.010	L85.0	242300
Neurofibromatosis	3	0.005	-0.001-0.010	Q85.0	162200
Pain insensitivity	2	0.003	-0.001-0.007		256800
Porphyria	2	0.003	-0.001-0.007	E80.20	176000
Adermatoglyphia	1	0.002	-0.002-0.005	Q82.8	136000
Alopecia	1	0.002	-0.002-0.005	L63	104000
Epidermolysis	1	0.002	-0.002-0.005	Q81.9	226650
Sebaceous naevus	1	0.002	-0.002-0.005	I78.1	162900
Vitiligo	1	0.002	-0.002-0.005	L80	606579
Xeroderma pigmentosum	1	0.002	-0.002-0.005	Q82.1	278700
***Others***	23	0.036	0.022-0.051		
Congenital heart defect	6	0.009	0.002-0.017	Q23.4	614954
Cardiac septal defect	3	0.005	-0.001-0.010	Q21.0	614429
Cleft lip/cleft palate	3	0.005	-0.001-0.010	Q37	119530
Infertility	3	0.005	-0.001-0.010		309120
Imperforate anus	2	0.003	-0.001-0.007	Q42.3	301800
Allergic rhinitis	1	0.002	-0.002-0.005	J30.9	607154
Type I diabetes	1	0.002	-0.002-0.005		
Hypothyroidism	1	0.002	-0.002-0.005	E03.1	275200
Lymphoedema	1	0.002	-0.002-0.005	I89.0	614038
Tetralogy of Fallot	1	0.002	-0.002-0.005	Q21. 3	187500
Tongue-tie	1	0.002	-0.002-0.005	Q38.1	

Among the neuromuscular disorders, cerebral palsy types had the highest representation (23.7%) (Table-II), followed by lower limb hypotonia and muscular dystrophy. Among neurological disorders, intellectual disability types had the highest representation (17%), followed by microcephaly. Among sensorineural/ear disabilities, deaf and mute cases (12.9%) had the highest representation. Among limb defects, talipes (3.1%) and polydactyly (2.2%) had the highest representation. While among the eye/visual impairments, blindness had the highest representation (2.4%) (Table-II).

### Parental consanguinity, familial/sporadic nature, and total affected family members:

Parental consanguinity of the overall cohort was 72%. The highest consanguinity was observed in sensorineural/ear defects (86%), followed by ectodermal anomalies (79%) and musculoskeletal defects (78%), while the lowest consanguinity rate was in the ‘others’ category (61%) (Table-III). The ‘Others’ category comprised less frequent anomalies (each <10% of total), including endocrine disorders, congenital heart defects, and cleft lip/palate.

**Table-III:** CA with respect to familial/sporadic nature, consanguinity, and total number of affected family members.

Major Division#	Total	Familial/sporadic nature		Parental consanguinity		Affected family members in all families		
		Familial	Sporadic	Yes	No	Male	Female	Total
Neuromuscular disorders	206 (32)	97 (47)	109 (53)	144 (70)	62 (30)	284 (67)	137 (33)	421 (30)
Neurological disorders	151 (24)	80 (53)	71 (47)	106 (70)	45 (30)	237 (64)	136 (36)	373 (26)
Sensorineural/ear defects	93 (15)	37 (40)	56 (60)	80 (86)	13 (14)	104 (65)	55 (35)	159 (11)
Limb defects	57 (9)	23 (40)	34 (60)	36 (63)	21 (37)	66 (63)	39 (37)	105 (7)
Eye/visual impairments	38 (6)	25 (66)	13 (34)	29 (76)	9 (24)	65 (50)	65 (50)	130 (9)
Musculoskeletal defects	27 (4)	16 (59)	11 (41)	21 (78)	6 (22)	34 (62)	21 (38)	55 (4)
Blood disorders	23 (4)	15 (65)	8 (35)	15 (65)	8 (35)	29 (59)	20 (41)	49 (3)
Ectodermal anomalies	19 (3)	11 (58)	8 (42)	15 (79)	4 (21)	45 (68)	21 (32)	66 (5)
Others	23 (4)	9 (39)	14 (61)	14 (61)	9 (39)	41 (63)	24 (37)	65 (5)
Total	637 (100)	313 (49)	324 (51)	460 (72)	177 (28)	905 (64)	518 (36)	1,423 (100)
		c^2^ =15.46, P=0.05		c^2^ =13.85, P=0.61		c^2^ =14.44, P=0.07		

#, values presented in number and (%)

Analysis of pedigree structures revealed that sporadic cases were 51% compared to 49% familial (Table-III). Familial occurrence was the highest in eye/visual impairments and blood disorders (66% and 65%, respectively), followed by musculoskeletal defects and ectodermal anomalies (59% and 58%, respectively). In contrast, the highest representation of sporadic cases was observed in sensorineural/ear and limb defects (60% each). In 637 families, there were a total of 1,423 affected subjects (905 males, 518 females) (Table-III).

### Ethnic and demographic differences in distribution of CA:

The distribution of major categories of CA was established across the demographic variables, and statistically significant differences were observed with respect to rural/urban origin, mother tongue, and caste system (Table-IV).

**Table-IV:** Ethno-demographic distribution of anomalies.

Variable#	Neuromuscular disorders	Neurological disorders	Sensorineural/ear defects	Limb defects	Eye/visual impairments	Others	Total
Rural/urban origin*							
Rural	157 (35)	104 (23)	50 (11)	42 (9)	30 (7)	68 (15)	451 (71)
Urban	49 (26)	47 (25)	43 (23)	15 (8)	8 (4)	24 (13)	186 (29)
Mother tongue*							
Saraiki	189 (34)	129 (23)	83 (15)	45 (8)	36 (6)	80 (14)	562 (88)
Pashto	12 (28)	12 (28)	1 (2)	8 (19)	2 (5)	8 (19)	43 (7)
Others^1^	5 (16)	10 (31)	9 (28)	4 (13)	0	4 (13)	32 (5)
Caste-system**							
Jaat	69 (53)	27 (21)	4 (3)	12 (9)	5 (4)	13 (10)	130 (20)
Pathan	26 (21)	32 (25)	18 (14)	16 (13)	9 (7)	25 (20)	126 (20)
Awan	32 (33)	22 (22)	16 (16)	7 (7)	6 (6)	15 (15)	98 (15)
Malik	22 (29)	19 (25)	19 (25)	3 (4)	3 (4)	10 (13)	76 (12)
Rajpoot	9 (19)	18 (38)	14 (30)	3 (6)	0	3 (6)	47 (7)
Baloch	18 (41)	9 (20)	2 (5)	2 (5)	8 (18)	5 (11)	44 (7)
Bhatti	14 (34)	7 (17)	7 (17)	6 (15)	3 (7)	4 (10)	41 (6)
Others	16 (21)	17 (23)	13 (17)	8 (11)	4 (5)	17 (23)	75 (12)

* P<0.05;

** P<0.0001; #, values presented in number and (%);

Other^1^ included minor language groups like Urdu, Punjabi and Sindhi.

Patients with neuromuscular disorders predominantly originate from rural areas and sensorineural defects from urban (p<0.05). With respect to the mother tongue, Saraiki- and Pashto-speaking individuals had a high representation of neuromuscular and neurological disorders, whereas neurological disorders and sensorineural defects were more common in ‘other’ language groups. Among the caste-systems, there was highest representation of neuromuscular disorders in Jatt, Awan, Malik, Baloch and Bhatti (53%, 33%, 29%, 41% and 34%, respectively); the representation of neurological disorders was highest among Pathan and Rajpoot (25% and 38%, respectively); the highest occurrence of sensorineural defects were among Rajpoot and Malik (30% and 25%, respectively); occurrence of limb defects was highest among Bhatti and Pathan (15% and 13%, respectively); Baloch had the highest representation of eye/visual impairments (18%) (Table-IV).

## DISCUSSION

Here, we report the first comprehensive study of ethno-demographic patterns and CA prevalence in the DIK region of Pakistan. Among the CA studied, neuromuscular disorders (32%), neurological disorders (24%), and sensorineural/ear defects were the most prevalent, a pattern that differs from previously reported prevalences estimates of CA in other parts of Pakistan. Such as, studies in Balochistan (27%), Peshawar (35%), and Hazara (41%) documented a higher occurrence of neurological anomalies.^9^,^10^,^6^ Similar trends were also reported in tertiary care hospital in Karachi,^8^ Azad Jammu and Kashmir,^11^ as well as in developing countries such as Brazil and India, where nervous system-related anomalies remained highly represented.^12^,^13^

A common neuromuscular anomaly in the present cohort is cerebral palsy (CP), particularly spastic CP with diplegia, as observed in national reports.^14^ Because CP is a non-progressive neurodevelopmental disorder, it is often accompanied by comorbidities such as intellectual disability (ID), epilepsy, and motor dysfunction, which further contribute to long-term disability. There was also a wide range of neuromuscular conditions present in this region, including lower limb hypotonia, muscular dystrophies, primary dystonias, and ataxias. Approximately 24% of the index cases had neurological disorders, with ID being the predominant type. These figures are somewhat lower than the 40% reported in Faisalabad 7 but align with studies carried out in Karachi, AJK, and Hazara, where ID constitutes a substantial proportion of the congenital burden.^8^,^11^,^6^ Studies have shown that neuromuscular and neurological disorders are influenced by genetic and environmental factors.

Remarkably, sporadic and familial cases were almost equally prevalent in our cohort. This contrasts with the data from Hazara (65%), and Peshawar (68%), where sporadic cases were more common.^6^,^10^ Sporadic cases may rise due to the rise of non-genetic environmental causes like poor antenatal care, maternal factors, nutritional imbalances, herbal intakes and taking unprescribed medicines. The predominance of neuromuscular disorders in rural areas may be linked to limited access to prenatal care, higher exposure to environmental toxins, or nutritional deficiencies. Conversely, higher sensorineural defects in urban settings could reflect better diagnostic access or differential effect of consanguinity.^15^

The parental consanguinity observed in this study (72%) is much higher than the national average consanguinity rate and is likely linked to the unusually high proportion of familial cases.^5^ High level of consanguinity observed in sensorineural/ear defects (86%), ectodermal anomalies (79%), and musculoskeletal defects (78%) may signify a strong role of autosomal recessive inheritance in these phenotypes.^4^,^16^

### Strengths of the study:

This study has many strengths. It reports a broad spectrum of CA prevalent in DIK population. At least one third of the anomalies observed in this cohort are significant contributors to morbidity and long-term disability, impacting an individual’s quality of life, necessitate prompt medical or surgical interventions to prevent further complications and improve outcomes. In addition to reporting prevalence metrics, our study provides valuable insights for public health policy making. By mapping the ethnic and geographical variations of CA, healthcare policies can be developed, community-based awareness campaigns can be built, genetic counseling programs can be offered, and targeted resources can be allocated. This study, however, does not report true prevalence rates of CA; further, the etiologies and molecular genetic diagnosis of CA were not reported.

### Limitations:

This study does not provide true community-based prevalence or incidence of CA. Due to constrained availability of advanced neuroimaging and electrophysiological tests, some disorders may have been misclassified or remained undiagnosed. Moreover, genetic bases of observed anomalies remain to be elucidated as molecular genetics and etiological diagnosis were not carried out.

## CONCLUSION

Our study highlights a high burden of neuromuscular and neurological disorders in the DIK region, with notable rural/urban and ethnic disparities. The coexistence of high parental consanguinity with a considerable proportion of apparently sporadic cases indicates that both genetic predisposition and environmental exposures shape the regional landscape of CA. These patterns reflect broader challenges faced by many resource-limited settings, where delayed health-seeking behavior, poor awareness, and fragmented health services contribute to underdiagnosis and late presentations. To reduce this burden, we recommend improved public health measures including community education programs, enhanced antenatal care, and premarital genetic counseling.

### Recommendations:

Future research should include prospective cohort studies to establish true prevalence, and molecular genetic screening to identify causative variants. It would be worthwhile to investigate the specific environmental risk factors prevalent in the DIK region which may be associated with certain types of CA.
